# Natural products: a hope for glioblastoma patients

**DOI:** 10.18632/oncotarget.25175

**Published:** 2018-04-24

**Authors:** Raghupathy Vengoji, Muzafar A. Macha, Surinder K. Batra, Nicole A. Shonka

**Affiliations:** ^1^ Department of Biochemistry and Molecular Biology, University of Nebraska Medical Center, Omaha, NE, 68198, USA; ^2^ Department of Otolaryngology/Head and Neck Surgery, University of Nebraska Medical Center, Omaha, NE, 68198, USA; ^3^ Eppley Institute for Research in Cancer and Allied Diseases and Buffett Cancer Center, University of Nebraska Medical Center, Omaha, NE, 68198, USA; ^4^ Department of Internal Medicine, Division of Oncology and Hematology, University of Nebraska Medical Center, Omaha, NE, 68198, USA

**Keywords:** glioblastoma, temozolomide, blood brain barrier and chemotherapeutic drugs

## Abstract

Glioblastoma (GBM) is one of the most aggressive malignant tumors with an overall dismal survival averaging one year despite multimodality therapeutic interventions including surgery, radiotherapy and concomitant and adjuvant chemotherapy. Few drugs are FDA approved for GBM, and the addition of temozolomide (TMZ) to standard therapy increases the median survival by only 2.5 months. Targeted therapy appeared promising in *in vitro* monolayer cultures, but disappointed in preclinical and clinical trials, partly due to the poor penetration of drugs through the blood brain barrier (BBB). Cancer stem cells (CSCs) have intrinsic resistance to initial chemoradiation therapy (CRT) and acquire further resistance via deregulation of many signaling pathways. Due to the failure of classical chemotherapies and targeted drugs, research efforts focusing on the use of less toxic agents have increased. Interestingly, multiple natural compounds have shown antitumor and apoptotic effects in TMZ resistant and p53 mutant GBM cell lines and also displayed synergistic effects with TMZ. In this review, we have summarized the current literature on natural products or product analogs used to modulate the BBB permeability, induce cell death, eradicate CSCs and sensitize GBM to CRT.

## INTRODUCTION

Tumors of the central nervous system (CNS) represent 1.4% of all newly diagnosed cancers and 2.6% of cancer deaths in 2015 [1]. Although rare, they are a significant cause of cancer morbidity and mortality and account for 30% and 20% of cancer related deaths in children and young adults respectively [1]. Brain tumors account for 85% - 90% of all primary CNS tumors. Glioblastoma (GBM) accounts for approximately half of all malignant adult brain tumors and is associated with the shortest survival [2]. Multimodality therapeutic intervention including surgery followed by adjuvant chemoradiation therapy (CRT) with temozolomide (TMZ), a DNA alkylating agent, is the standard of care for GBM. The addition of TMZ increased the overall survival (OS) from 7.7 to 13.5 months and from 7.9 to 10.0 months in the GBM patients with methylated and non-methylated O^6^-methylguanine-DNA methyl transferase (MGMT) respectively [3, 4]; however survival remains very poor. This poor survival is likely a product of many factors, including systemic toxicity of higher TMZ doses, BBB impermeability, resistance to CRT and development of refractory tumors [5]. Therefore, there is a crucial need for identifying novel compounds that are able to modulate the BBB, inhibit tumor growth and prevent development of recurrent tumors for improved overall patient prognosis.

In the last two decades, natural product based therapy has gained popularity as effective and potentially less toxic treatment. Roots of *Podophyllum peltatum* (mayapple) were used by the American Indians long ago to treat many skin cancers [6]. The principal anticancer constituent podophyllotoxin and its semisynthetic derivatives, namely Teniposide, Etoposide and Etopophos are extensively used to treat several cancers [7]. The National Cancer Institute (NCI) initiated two mega- scale anti-cancer drug-screening programs during 1960 and 1985. From that screening, they identified an important compound Taxol (paclitaxel), isolated from the bark of *Taxus brevifolia* that has since been used to treat many solid tumors. Moreover, nearly one-third of the drugs approved by the United States Food and Drug Administration (USFDA) for cancer are from natural products or their analogs [6, 8]. We have summarized the published literature on natural products and their analogs that have been used to treat GBM using *in vitro* and *in vivo* models. In addition, we also discuss the utility of many natural compounds including procyanidine and scillarenin in modulating the BBB to improve drug delivery and enhance therapeutic efficacy.

## NATURAL PRODUCTS AND GBM

GBM represents a highly invasive and highly heterogeneous type of malignant brain tumor [9]. Detailed molecular analysis of GBM reveals dysregulation of core signaling pathways including those that regulate cell growth, DNA repair and apoptosis like receptor tyrosine kinase (RTK), phosphoinositide 3-kinase (PI3K) signaling, mitogen activated protein kinase (MAPK) signaling, retinoblastoma and p53 signaling [9]. In addition, 30–40% of GBM patients have mutations in the tumor suppressor gene TP53 [10] resulting in chemo- and radio- resistance. TP53 encodes for p53, a transcription factor known to regulate multiple functions such as DNA repair, cell cycle arrest, senescence, apoptosis and metabolism. Haas-Kogan *et al.*, observed increased radio-resistance to fractionated radiation therapy (RT) in GBM cells expressing mutated p53 [11], while transduction of wild type p53 in the 9L GBM cell line increased sensitivity to cisplatin [12]. Although TMZ is BBB permeable and is less myelotoxic than other drugs available for GBM [13], unfortunately, mutant TP53 confers TMZ resistance by up-regulating MGMT expression in T98G and U138 GBM cell lines [14]. While TMZ induces DNA damage by methylating the O^6^ position of the guanine base in DNA, active MGMT (22 kDa protein) rapidly removes methyl groups than other alkyl groups linked to the O^6^ position of guanine [15] and directly repairs the TMZ-damaged DNA [16]. Although the current TMZ based CRT has marginally improved survival in MGMT methylated (ie MGMT inactivated) GBM patients, its cytotoxicity is relatively nullified in unmethylated GBM patients [17]. Recent, recent analysis of quantitative methylation using pyrosequencing on 108 GBM patients revealed that degree of MGMT promoter methylation is directly associated with median progression free survival [18]. Currently, small molecule inhibitors targeting MGMT are being utilized prior to TMZ based therapy in many clinical trials [19]. In addition, research is focused to identify minimally toxic compounds that are able to target novel deregulated signaling pathways, evade the BBB, and enhance therapeutic efficacy. Plant based products have long been used to influence cancer development, progression, and metastasis. A number of studies have revealed the antitumor potential of natural compounds used either alone or in combination with chemotherapy (CT) and RT in GBM and are summarized below.

### Natural products as chemosensitizers in GBM

The efficacy of CRT for GBM is limited by poor drug availability, treatment toxicity, and chemoradiation resistance. Natural products and product analogs with potential as chemo/radio sensitizers in GBM are summarized in Table 1.

**Table 1:** Natural products and product analogs with potential as chemo/radio sensitizers in GBM

S. No	Scientific Name	Component	Function	Refs
1	*Allium cepa*	Quercetin	↓ Hsp27 and ↑ TMZ sensitivity in U87 and U251 GBM cell lines	[28]
2	*Vitis vinifera*	Resveratrol	↓MGMT expression, ↓Nf-ƘB signaling and ↓ antiapoptotic proteins XIAP and survivin and ↑TMZ sensitivity in T98G GBM cells	[32]
3	*Vitis vinifera*	Resveratrol	↑ROS generation, ↑AMPK activation , ↓ mTOR signaling , ↓ antiapoptotic protein Bcl-2 and ↑TMZ sensitivity in SHG44 GBM cells; ↓orthotopic GBM xenograft with TMZ	[33]
4	*Herba Epimedi*	Icariin	↓ proliferation of U87 GBM cells; ↓ Nf-ƘB signaling , ↓migration, ↓invasion and ↑TMZ sensitivity in U87 GBM cells	[37]
5	*Ficus carica*	Latex	↓ proliferation of U87, U138MG and T98G GBM cell lines; ↑ tumor suppressor let-7miRNA, ↓invasion and ↑TMZ sensitivity	[42]
6	*Apis mellifera*	Ethanolic extract of Propolisis	↓Nf-ƘB signaling and ↑ TMZ sensitivity in U87 GBM cells	[43]
7	*Apis mellifera*	Propolisis	↓ cell proliferation in U343 and U251 GBM cell lines, ↑ chemosensitivity to TMZ	[49]
8	*Zataria multiflora*	Hydroalcoholic extract	↑ radiosensitivity of A172 GBM cells	[86]
9	*Stephania* *tetrandra* S. Moore	Tetrandrine	↑G_0_/G_1_ cell cycle arrest; ↓ radiation induced ERK signaling and proliferation associated genes PCNA and CCND1; ↑ radiosensitivity of U87 and U251 cell lines	[88]
10	*Withania somnifera*	Withaferin A	↓ cell proliferation of U87, U251 and TMZ resistant GBM cell lines U87 TMZ, U251 TMZ, T98G and U138 in a concentration dependent manner, ↑ TMZ sensitivity by ↓ MGMT expression.	[53]
11	*Curcuma longa*	Turmeric Force^™^	↑ sensitivity to TMZ in U87 GBM cells	[204, 205]

### Quercetin

Cancer induced inflammation can accelerate tumor cell proliferation, survival and migration. Interleukin-6 (IL-6) is the primary cytokine which creates the inflammatory peri-tumoral environment. Its increased expression in GBM [20] is directly associated with poor patient survival [21]. In addition, persistent activation of Signal Transducer and Activator of Transcription - 3 (STAT3) by autocrine expression of IL-6 is observed in GBM cell lines. Scavenging of IL-6 using specific antibodies repressed cell proliferation and stimulated apoptosis [22]. Quercetin, a natural flavonoid ubiquitously present in various vegetables and fruits including broccoli, red onions, apples, red grapes, cherries and berries [23] has been identified as an antioxidant and anticancer agent [24–26]. Specific to GBM, Jonathan *et al.*, showed that quercetin treatment significantly decreased the IL-6 mediated STAT3 activation in U87 and T98G cell lines in a concentration dependent manner [27]. This flavonoid also increased the sensitivity of GBM lines U87 and U251 to TMZ by suppressing heat shock protein 27 (Hsp27) expression, that is known to confer drug resistance [28]. Additionally, quercetin has also been shown to induce mitochondria mediated apoptosis in the resistant p53 mutant GBM cell line U373MG [29]. In contrast to these anti-tumorigenic properties of Quercetin, pro-tumorigenic effects of Quercetin were also reported in a rat glioma model [30].

### Resveratrol

**Figure 1:**
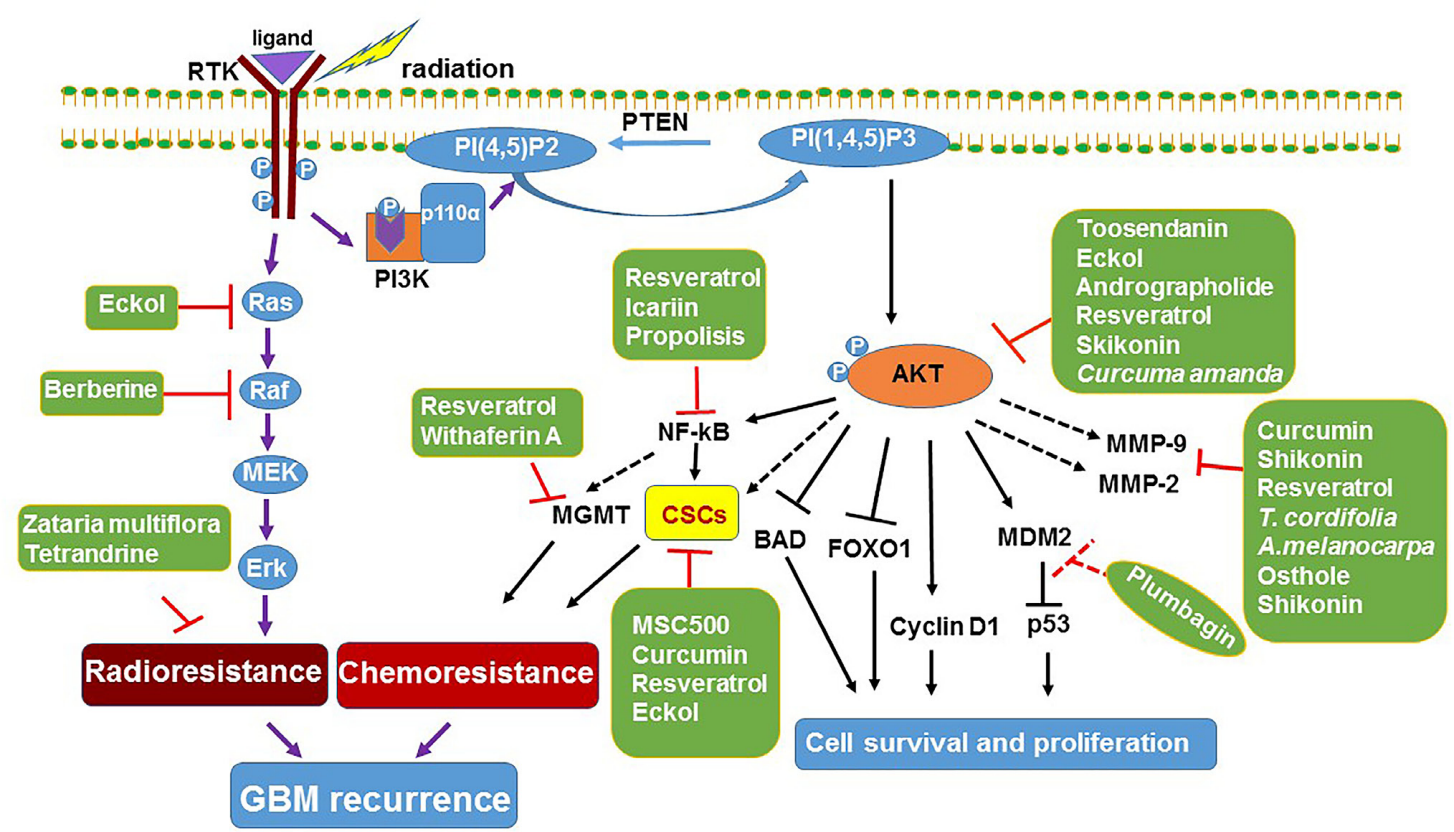
Mechanism of natural product based sensitization of GBM RTKs activate Ras/Raf/MAPK signaling cascade to increase GBM cell proliferation. The RTK/PI3K/AKT signaling pathway inhibits apoptosis by phosphorylating the pro-apoptotic protein BAD resulting in its cytoplasmic sequestration to inhibit cytochrome-C release from mitochondria. It also inactivates the transcription factor FOXO1 by phosphorylation, resulting in inactivation of pro-apoptotic proteins and cell survival. Natural products resveratrol and eckol attenuate both the Ras/Raf and PI3K/AKT signaling pathways, inhibit cell proliferation, induce apoptosis and also eradicate GBM CSCs; Korean natural medicine recipe, MSC500 decreases CSCs population by decreasing the aldehyde dehydrogenase (ALDH) activity and downregulated the expression of ABC transporters (ABCG2 and ABCB5). Moreover, natural products especially resveratrol and Withaferin A increased the TMZ sensitivity by downregulating MGMT expression. *Zataria multiflora* hydroalcoholic extract and tetrandrine significantly increased the radiosensitivity of the GBM cell lines. RTK, receptor tyrosine kinase; MAP, mitogen- activated protein kinase; CSCs, cancer stem cells; PTEN, phosphatase and tensin homolog; MGMT, O^6^-methylguanine-DNA methyl transferase; PI (4,5)P2, phosphatidylinositol-4,5-bisphosphate; PI(1,4,5) P3 - phosphatidylinositol-3,4,5-trisphosphate. Dotted arrow indicates expected effects.

Resveratrol is a potent anti-oxidant found in grapes, peanuts and mulberries [31] with known anti-tumor activity [31]. Huang *et al.*, demonstrated that resveratrol treatment significantly decreased TMZ resistance by downregulating the expression of MGMT, at least in part through NF-ƘB dependent signaling in T98G GBM cells [32] (Figure 1). It also significantly decreased the expression of the anti-apoptotic proteins, X-linked inhibitor of apoptosis protein (XIAP) and survivin [32]. Another study showed that resveratrol increased TMZ toxicity by increasing reactive oxygen species (ROS) generation, AMPK pathway activation, mTOR signaling inhibition and decreased antiapoptic protein Bcl-2 expression in SHG44 GBM cells [33]. Furthermore, combination treatment of resveratrol with TMZ also significantly reduced the orthotopic xenograft growth of GBM cells [33].

### Icariin

Icariin is a flavonoid extracted from the Chinese medical herb *Herba Epimedi*. Icariin is known to have cardio-protective, anti-inflammatory, bone-healing, anti-depressant, neuro-protective and anti-cancer properties with low toxicity [34, 35]. It is believed to cross the BBB [36] and therefore can be more available to the tumor cells. Lijuan *et al.*, identified that icariin treatment significantly decreased the growth of U87 GBM cells in a concentration dependent manner [37]. He further showed that Icariin augments the cytotoxicity of TMZ and decreased the migration and invasion of U87 GBM cells probably by attenuating NF-ƘB activity [37].

### Latex and resins

MicroRNAs (miRNAs) are small non-coding RNA molecules that regulate gene function during transcription and translation and play both pro- and anti-tumorigenic roles. Let-7 miRNA is one of the important miRNAs that suppress tumor growth and is downregulated in many cancers [38–40]. Overexpression of Let-7 sensitized cancers to the chemotherapeutic agent cisplatin [41]. Recent studies have shown that latex from the *Ficus carica,* a member of the mulberry family significantly inhibited the proliferation of U87, U138MG and T98G cells by upregulating let-7 expression [42]. Furthermore, it attenuated cell invasion and induced TMZ sensitivity by upregulating let-7 expression [42]. Propolis resin from the honeybee is comprised of flavonoids, steroids, terpenes, vitamins (B1, B2, C and E), esters and sugars [43]. While propolis is well documented to have antibacterial, antiviral, antifungal, and immunomodulatory functions [44–47], a recent study showed anti-cancer activities from its flavonoid components [48]. Kleiton *et al.*, showed that propolis decreased the proliferation of U343 and U251 GBM cells and human lung fibroblast cell line MRC-5 [49]. More interestingly, they showed a synergistic antiproliferative effect of propolis in combination with TMZ in GBM cells [49]. Renata *et al.* also demonstrated that an ethanolic extract of propolis in combination with TMZ inhibited the growth of U87 cells [43]. Finally , they showed that the antiproliferative effect of propolis was due to NF-ƘB [43] inhibition, which is known to play a vital role in GBM [50].

NF-ƘB signaling may alter TMZ sensitivity [32, 37, 43], at least in part by downregulating MGMT expression [32]. The regulation of NF-ƘB and its activity is mainly controlled by PI3K/AKT signaling [51, 52]. Interestingly, the natural compound Withaferin A mediated MGMT downregulation and the resultant TMZ sensitivity was associated with inhibition of the EGFR/AKT/mTOR signaling pathway [53]. Surprisingly, PI3K/AKT signaling has also been shown to play a vital role in GBM radioresistance [54, 55]. Plant derived products known to inhibit PI3K/AKT signaling pathways may be used as a chemo- and radio- sensitizers are summarized below.

### Bittersweet

*Celastrus orbiculatus,* commonly known as bittersweet belongs to the Celastraceae family and is used as a folk medicine to treat numerous diseases including rheumatoid arthritis. Celastrus and its several constituents have shown to possess anti-oxidant, anti-inflammatory and anti-cancer properties [56]. Recently, *Celastrus orbiculatus* extract (COE) was shown to inhibit cell proliferation, adhesion and migration of human gastric cancer [57, 58], and induced apoptosis and autophagy in colorectal cancer cells by modulating the PI3K/Akt/mTOR signaling pathway [56]. Hao *et al.* also demonstrated decreased cell viability, adhesion, migration and invasion of U87 and U251 GBM cells by COE [59]. While this study didn’t directly analyze the effect of COE on the PI3K/Akt/mTOR signaling pathway, the involvement of this pathway in regulating invasion and motility in both GBM cell models suggest that COE may be inhibiting the PI3K pathway. Further mechanistic studies are needed.

### Andrographolide

*Andrographis paniculata* (AP) is a medicinal herb generally known as Kalamegha or Kalmega and widely distributed in India. Andrographolide, a bicyclic diterpenoid lactone isolated from the leaves of AP has been shown to possess anti-cancer activity against many tumors and was also shown to cross the BBB [60, 61]. Li *et al.*, demonstrated that andrographolide inhibits U87 and U251 GBM cell proliferation by inducing G_2_/M cell cycle arrest, accompanied by decreased expression of proteins Cdk1 and Cdc25C [61]. They also showed inhibition of the PI3K/AKT/mTOR signaling pathway in U87 and U251 cells by andrographolide [61].

### Plumbagin

Plumbagin is a bicyclic naphthoquinone that is present in the roots of Droseraceae, Plumbaginaceae and Ebenceae family members. It belongs to one of the widespread and diverse groups of plant metabolites. This natural pigment has antidiabetic, antioxidant, antimutagenic, anti-inflammatory and anti-proliferative properties against leukemia, melanoma, lung, breast, and prostate cancer [62–67]. The inhibitory effect of plumbagin was shown by modulating several signaling pathways including Akt/mTOR, NF-kB, and JNK. Recently, its effect was investigated on GBM cells and it was observed that plumbagin induced cell cycle arrest and DNA damage followed by apoptosis [68]. The mechanistic studies revealed upregulation of TNFRSF1A, PTEN and downregulation of E2F1 genes, MDM2, cyclin B1, survivin and Bcl2 protein expression along with increased caspase-3/7 activity. Strikingly, they observed that plumbagin inhibits telomerase activity and shortening of telomeres upon chronic plumbagin exposure [68]. Furthermore, they observed enhanced cytotoxicity of plumbagin to KNS60 GBM cells with higher telomerase activity than in U251 and A172 GBM cells with less activity [68].

## NATURAL PRODUCTS AS GBM RADIOSENSITIZERS

RT alone or in combination with TMZ is the standard of care for most GBM patients. However, toxicity to normal tissues and development of resistance limits the efficacy of RT [69]. Therefore, agents that can radiosensitize tumor cells would not only prevent the development of resistant tumors, but may also help to reduce RT associated toxicity. Many synthetic and naturally derived compounds have been used as radiosensitizers for GBM. However due to the intrinsic immune enhancing properties of many natural products, they enhance RT effects with less toxicity to normal tissues. Natural compounds used or potentially to be used as radiosensitizers in GBM are discussed below.

### Guduchi

*Tinospora cordifolia,* commonly called Guduchi, belongs to the family Menispermaceae. It has been used in ayurvedic treatment for centuries to treat jaundice and to protect liver function [70–72]. Its anti-angiogenic, anticancer, anti-inflammatory and radiosensitizing properties are also well documented [73–78]. Rao *et al*., demonstrated Guduchi’s radiosensitizing activity in ehrlich ascites carcinoma (EAC) mice [79]. Interestingly, animals pre-treated with 30 mg/kg guduchi extract 1 hour before 6 Gy of γ-radiation and subsequent guduchi treatment for 6 days reduced the tumor growth and increased the overall survival of EAC mice compared to animals without pre-treatment [79]. Ethanolic extract of Guduchi EEG also inhibited the growth of C6 rat glioma cells and U87 GBM cells in a concentration dependent manner and also induced differentiation of C6 cells to an astrocyte-like phenotype [80]. In addition, EEG inhibited the migration and invasion of C6 cells associated with decreased matrix metalloproteinases -2 and -9 (MMP-2 and MMP-9), NCAM and PSA-NCAM expression [80]. Furthermore, EEG-induced cell cycle arrest and senescence was accompanied by decreased expression of cyclin D1, Bcl-xL and increased expression of mortalin, a marker for senescence [80].

### Zataria multiflora

*Zataria multiflora* is known as Avishan-e-Shirazi in Iran and belongs to the Lamiaceae family. In addition to containing a small percentage of saponins, caffeic acid, resin, tannin, and resonates, zataria extract (ZE) contains 69% phenols-primarily carvacrol' P-cymene and thymol [81]. Many recent studies showed that ZE possesses anti-bacterial, anti-oxidative and anti-inflammatory activity and most importantly protected lymphocytes from radiotherapy [82–85]. More specifically, hydroalcoholic extract (200 ug/ml) of this plant significantly increased the radiosensitivity of A172 GBM cells in a concentration dependent manner and induced apoptosis [86]. Interestingly, ZE showed no antiproliferative or radiosensitizing effects in nonmalignant HFFF2 cells [86] suggesting its nontoxicity to the normal cells.

### Tetrandrine

Tetrandrine (Tet) is a bisbenzylisoquinoline alkaloid that is isolated from the roots of *Stephania tetrandra S. Moore*, with known apoptotic, anti-angiogenic and radiosensitizing properties in many cancers [87, 88]. Recently, Tet was also shown to radiosensitize GBM cells U87 and U251 by significantly inhibiting their proliferation via inducing G_0_/G_1_ cell cycle arrest, attenuating the radiation induced ERK signaling pathway and decreased expression of proliferation associated genes PCNA and cyclin D1 [88].

## NATURAL PRODUCTS EFFECTING GBM CELL PROLIFERATION AND APOPTOSIS

GBM is characterized by uncontrolled proliferation, local necrosis, diffuse infiltration, and increased angiogenesis [89]. In addition, like many other cancers, GBM cells are relatively resistant to apoptosis due to deregulation of pro- and anti-apoptotic proteins [90, 91]. Natural products that inhibit proliferation and induce apoptosis in GBM cells are summarized below.

### Alkaloids and flavonoids

*Zingiber officinale* and *Rhazya stricta* belong to the Apocynaceae and Zingiberaceae families respectively. Alkaloids extracted from *Rhazya stricta* have antimicrobial and anticancer properties and chemosensitize many tumors [92–94]. While the flavonoids are known to exhibit anti-angiogenic, pro-apoptotic and anti-cancer activities in *in vitro* and *in vivo* models [95, 96], effects of flavonoids of *Zingiber officinale* on GBM cells have not been explored extensively. Recently, Ayman *et al.*, studied the effect of crude flavonoid and alkaloid extract of *Zingiber officinale* and *Rhazya stricta,* respectively, on a U251 GBM cell line. They observed that this combination resulted in synergistic growth inhibition, decreased clonogenic survival and induction of apoptosis [97]. They showed the induction of apoptosis was mediated by cytochrome c release from mitochondria, increased Bax/Bcl-2 ratio, caspase 3/9 activation and cleavage of poly (ADP-ribose) polymerase (PARP). Additionally, the crude extract induced apoptosis and was associated with decreased nuclear NF-κB, expression of survivin, XIAP, cyclin D1 and increased p53, p21, and Noxa expression [97].

### Oridonin

Oridonin is diterpenoid compound extracted from the Chinese herbal medicinal plant *Rabdosia rubescens*. Oridonin was found to have anti-cancer activities against multiple cancers including breast, lung, leukemia and osteosarcoma [98, 99]. Recently, oridonin mediated inhibition of RanGTPase activating protein 1 (RanGAP1) was shown to induce apoptosis in U87 GBM cells by effecting nuclear cytoplasmic export of noncoding RNA (ncRNA) [100]. It is important to mention that tumor cells synthesize relatively very high ncRNAs and RanGAP1 is believed to be an important protein involved in nucleo-cytoplasmic export of ncRNA *via* the nuclear pore complex (NPC).

### Osthole

Osthole or osthol is a natural coumarian, extracted from ripe cnidium fruits and known to have anti-oxidant, anti-inflammatory and anti-cancer properties [101–103]. Recently, Kai *et al.* have shown that osthole treatment significantly reduced growth, enhanced apoptosis and increased the expression of the tumor suppressor microRNA-16 (miRNA-16) with decreased MMP-9 expression in U87 GBM cells [104]. Of note, immunohistochemical analysis in GBM revealed that MMP-9 expression is increased in GBM when compared to normal brain [105]. siRNA mediated silencing of MMP-9 resulted in decreased GBM proliferation and increased apoptosis [106, 107]. These studies suggest that osthole induced apoptosis may be mediated through MMP downregulation, which further needs to be explored.

### Cucurbitacin

Cucurbitacins are terpene sterols that are extracted from the Cucurbitaceae family of plants and structurally classified into 12 groups [108]. Among them, cucurbitacins I, E, B, D and Q are well accepted for their anti-neoplastic activity in several cell lines [109–112]. The anticancer effects of cucurbitacins involve induction of cell cycle arrest and apoptosis by inhibiting the Janus kinase/Signal Transducer Activator of Transcription 3 (JAK/STAT3) signaling pathways. Dong *et al.* also reported that cucurbitacin B repressed proliferation and colony formation of U87 and T98G GBM cells. Further, cucurbitacin B disrupted actin and the microtubule network, resulting in loss of pseudopodia thereby inhibiting cell migration/invasion [113].

### Chokeberry extract and curcumin

Chokeberry (*Aronia melanocarpa*) belongs to the Rosaceae family and is distributed in eastern North America. Polyphenolic extract of chokeberry (CPE) contains several flavonoids and anthrocyanins thought to penetrate the BBB [114]. Azela *et al.* reported recently that both CPE and curcumin decrease the viability of U373 GBM cells [115]. While CPE decreased viability by inducing necrosis, curcumin directly induced apoptosis in U373 GBM cells [115]. Furthermore, both of these compounds downregulated MMP-2, MMP-14, MMP-16 and MMP-17 mRNA levels [115]. Since MMPs are an integral part of the BBB and maintain its integrity, curcumin and CPE mediated decrease of MMP’s may modulate BBB permeability and may enhance drug diffusion.

### Jaceosidin

*Artemisia argyi* (AA), generally known as silvery wormwood, belongs to the Compositae family and is distributed throughout the world. Muhammad *et al.* isolated Jaceosidin from the leaves of AA and analyzed its anti-cancer activity on U87 GBM cells [116]. He observed that Jaceosidin treatment inhibited proliferation, induced cell cycle arrest in the G_2_/M phase and promoted apoptosis. This increased apoptosis was accompanied by an increase in the proapoptotic protein Bax and p53 expression, and abolished the mitochondrial membrane potential (MMP), cytochrome C release to cytoplasm and activation of caspase-3 [116].

### Trichosanthin

Trichosanthin (TCS) belongs to the family of plant proteins known as ribosome-inactivating proteins (RIPs) and is isolated from the roots of the herb *Trichosanthes kirilowii, generally* known as Chinese snake gourd and Chinese cucumber. TCS is traditionally used in Chinese medicine for inducing midterm abortion [117]. By virtue of its rRNA N-glycosidase activity, it attacks the eukaryotic cell ribosomes and inhibits protein translation [117]. TCS exhibits antitumor effects against cervical cancer, choriocarcinoma, leukemia and lymphoma. In addition, TCS has also been shown to significantly inhibit proliferation of U87 and U251 cells [118] and increase apoptosis in U87 cells by decreasing the leucine-rich repeat containing G-protein coupled receptor 5 (LGR5) and Wnt/β-catenin expression [118]. Importantly, LGR5 expression positively correlates with increasing histologic grade of astrocytoma – from grade II to GBM (grade IV) – and is associated with poor survival [119]. LGR5 mRNA levels are also higher (10 fold) in the GBM CSCs than healthy brain astrocytes. Predictably, adenovirus mediated silencing of LGR5 induced cell death in GBM CSCs [119].

### γ-Mangostin

γ-Mangostin is a xanthone derived from *Garcinia mangostana*, commonly known as mangosteen “the queen of fruit”. Hui-Fang *et al.* reported significant inhibition of growth and apoptosis of U87 and 8401 GBM cells in a concentration dependent manner by γ-Mangostin [120]. Further, the biochemical investigation revealed that γ-Mangostin induced apoptosis was associated with increased hypodiploid cells, mitochondrial dysfunction and increased ROS production [120]. In addition to GBM, γ-Mangostin also showed anti-proliferative effects on human colon cancer DLD-1 cells [121].

### Thymoquinone

Thymoquinone (TQ), is the major bioactive compound extracted from *Nigella sativa* seed, commonly known as black seed and is vastly distributed in India, Eastern and European countries. TQ is known for its anti-cancer activities against several cancer cell lines with low toxicity to normal cells [122–124]. Recent study has shown inhibition of proliferation of Gli36EGFRvIII, T98G and U87 GBM cells by TQ [125]. They also observed that this decrease in cell proliferation was independent of p53 status and may occur through inhibition of autophagic flux [125]. In addition to GBM, our lab has also shown strong antitumor effects of thymoquinone against pancreatic cancer [126].

### Brazilin

Brazilin is a red pigment extracted from the central wood of the *Caesalpinia sappan* with known anti-oxidant, anti-inflammatory and anti-proliferative properties [127]. In addition, Dae-Young also demonstrated that brazilin also decreased proliferation and induced apoptosis in U87 GBM cells as shown by the cell cycle arrest at sub-G_1_ phase, while decreasing the expression of caspase -3 and caspase-7, and increasing the expression of PARP [127].

### Betulinic acid

Betulinic acid (BetA), is a pentacyclic triterpenoid extracted from birch trees (*Betula pubescens*) and selfheal (*Prunella vulgaris*). Besides anti-retroviral, anti-malarial, and anti-inflammatory properties, recent studies have shown it to exhibit potent anti-cancer activity against several human cancers with no significant effect on normal cells [128–130]. Interestingly, it was shown to induce apoptosis in human neuroblastoma cells *in vitro* and *in vivo* [131]. In addition, by activating a p53 independent caspase - PARP cascade, BetA induced apoptosis in five glioma cell lines [132]. BetA induced cell death was associated with increased levels of the proapoptotic protein BAX, formation of ROS, and DNA fragmentation [132]. Similarl observation of BetA induced cell death was also reported by Simone. *et al* using a large panel of malignant brain tumor cells [133].

### Deoxypodophyllotoxin

Deoxypodophyllotoxin (DPT) is a semi-synthetic compound isolated from *Dysosma versipellis* extract. Mounia *et al.* identified that DPT treatment significantly inhibited the proliferation coupled with G_2_/M cell cycle arrest in U87 and SF126 GBM cells at nanomolar concentrations [134]. It decreased the expression of cell cycle regulatory proteins including cyclin B1, Cdc2 and Cdc25C in U87 GBM cells [134]. In addition, DPT treatment also induced apoptosis in both U87 and SF126 cells by abolishing the MMP, while decreasing the expression of Bcl-xL and Bcl-2 [134].

### Nardostachys jatamansi

*Nardostachys jatamansi* (NJ) commonly known as spikenard, nardin, nard or muskroot belongs to the Caprifoliaceae family and is found at high altitudes in India. Crude extracts from the NJ are effective against parkinsonism and epilepsy in experimental brain models [135, 136]. A methanol extract (ME) of NJ rhizome significantly inhibited the proliferation of U87 GBM cells with less cytotoxicity to human embryonic kidney (HEK) cells, suggesting its relative safety to normal cells. ME also induced DNA fragmentation coupled with apoptosis in U87 cells [137].

### Variolin B and Meridianins

Uncontrolled proliferation due to sustained activation of cyclin-dependent kinases (CDKs) is the hallmark of most cancers, leading to the widespread investigation of inhibitors specific to CDKs. The cyclin D-CDK4/CDK6 signaling pathway is deregulated in GBM and results in uncontrolled cell cycle progression [138, 139] making it a viable targeted therapeutic option. Natural compounds like Variolin B and Meridianins isolated from the marine Ascidian *Aplidium meridianum* inhibit CDK activity [140]. Recently, synthetic hybrid molecules called meriolins were derived from meridianins, and shown to induce apoptosis in neuroblatoma SH-SY5Y cells [141]. Comparative analysis of a panel of meriolins showed that meriolin-15 significantly inhibited proliferation and induced apoptosis in GBM cells [142].

### Xanthones and Lactones

Xanthones belong to class of tricyclic compound with known anti-cancer, anti-microbial and anti-inflammatory activities [143–145]. The Cudraxanthone-I xanthone extracted from *Milicia excelsa* significantly inhibited the proliferation of the U87 and resistant U87 EGFRvIII GBM cell lines [146]. Lactones *derived from the Vernonia cinerea* (little ironweed or ash fleabane) of the Asteraceae family have antimalarial, anti-inflammatory and anti-metastatic properties [147–149]. Sesquiterpene lactones isolated from *Vernonia cinerea* significantly inhibited STAT3 activity in U251 GBM cells and decreased their viability [150].

### Salidroside

Salidroside or rhodioloside, a glucoside of tyrosol, is obtained from root extracts of *Rhodiola crenulata.* Salidroside exhibits anti-cancer activities against gliomas, gastric, breast and lung cancers [151]. Similarly, purified salidroside also inhibited proliferation and induced G_0_/G_1_ cell cycle arrest of glioma U251 cells [152]. Crude extract from the root (CER) also decreased proliferation and clonogenic survival of U87 GBM cells [153]. Of note, CER mediated decrease in cell proliferation was associated with inhibition of Wnt/β-catenin signaling and enhanced glial fibrillary acidic protein (GFAP) expression [153].

### Rutin

Vascular endothelial growth factor (VEGF) and transforming growth factor-β1 (TGF-β1) are the two major cytokines recognized to influence GBM cell migration, invasion and angiogenesis [154, 155]. Rutin, a flavonoid isolated from seeds of *Dimorphandra mollis* , is commonly known as faveira. Interestingly, rutin was shown to inhibit the production of both VEGF and TGF-β1 in GBM GL-15 cells and therefore, inhibit angiogenesis and CRT resistance [155]. It is interesting to note that the anti-VEGF antibody bevacizumab suppresses VEGF production but doesn’t affect TGF-β1 expression or activation [155]. Rutin is known to inhibit skin cancers as well [156].

### Lichen derivative

Lichens are complex organisms, made of fungal filaments and one or more cyanobacteria or photosynthetic algae. Protolichesterinic acid, lobaric acid, usnic acid and vulpinic acid isolated from lichens were found to have anti-proliferative, anti-oxidant, and antibiotic activities [157, 158]. The methanolic extracts (ME) of lichens *Cladonia rangiformis* and *Cladonia convolute* significantly inhibited proliferation of the GBM cell line U251 [159]. Recently, it was also reported that olivetoric and psoromic acid from lichens significantly reduce the viability of U87 GBM cells possibly by inducing oxidative DNA damage [160].

### Cactus

*Opuntia humifusa,* generally known as the devil’s tongue, belongs to the Cactaceae family [161, 162] and its extracts display antibacterial and anti-oxidant effects [163]. While the ethyl acetate extract (EAE) of cactus show cytotoxicity against colon, cervical and breast cancer cells, hexane extract (HE) and water partitioned fraction (WPF) decreased proliferation of U87 GBM cells [163]. This WPF induced decrease in cell proliferation was associated with G_1_ phase cell cycle arrest, enhanced ROS generation and non-apoptotic cell death [163].

### Diosquinone

Diosquinone, a napthoquinone epoxide, is extracted from the root bark of *Diospyros mespiliformis* (Hostch) and Ebenaceae *Diospyros tricolor*. Recent study has shown that Diosquinone inhibited cell proliferation of many cancer cells including GBM [164]. They further reported greater cytotoxicity of Diosquinone against p53 mutant GBM cell line U373 than lung, colon, prostate and neuroblastoma cancer cell lines [164].

### Tithonia diversifolia

Survivin is a member of anti-apoptotic gene family whose expression is elevated in most solid tumors with undetectable to minimal expression in normal and differentiated cells [165]. Overexpression of survivin is associated with tumor recurrence and drug resistance [166–168]. The medicinal herb *Tithonia diversifolia,* generally known as Mexican sunflower or Japanese sunflower, has known anticancer activities against human colon cancer Col2 cells and human promyeleocytic leukemia HL-60 cells [169]. In addition, crude *Tithonia diversifolia* extract and its principal compound tagitinin C also inhibited growth of U373 GBM cells by downregulating survivin expression in a dose dependent manner [170].

### Zeng sheng ping

Zeng Sheng Ping (ZSP) is a Chinese herbal mixture containing *Polygonum bistorta*, *Sonchus brachyotus*, *Dioscorea bulbifera*, *Sophora tonkinensis*, *Prunella vulgaris* and *Dictamnus dasycarpus*. Kah *et al.*, reported that ZSP inhibited proliferation and survival of U87 cells possibly by downregulating Notch2 and its signaling component Hes1 [171]. ZSP also decreased expression of the stem cell markers CD133 and nestin in U87 cells [171]. Notch signaling is highly activated in GBM and targeting this pathway prevented tumor growth and improved survival [172–175]. In addition, many pre-clinical studies showed antitumor effects of ZSP against colon and esophageal cancers [176–178]. More importantly, many clinical studies also showed that ZSP significantly prevented the progression (48–52 %) of esophageal cancers [179–181].

### Tanacin

*Tanacetum huronense* is commonly known as Lake Huron tansy. Ethyl acetate extract of this plant and its bioactivity based purification have yielded six sequiterpenoid lactone compounds. Among them, compound 4 (tanacin) displayed highest inhibitory effect against the U87 GBM cell line [182].

### Procyanidins

Procyanidins, a class of flavonoids, are oligomeric compounds formed from catechin and epicatechin molecules. They are mostly present in grape seeds, grape skin, apples, cinnamon, cocoa beans etc. These procyanidins can be grouped into 7 fractions (F1 to F7) depending upon their degree of polymerization. Recent studies have shown that F2, after crossing the BBB, protected mouse brains from ethanol induced oxidative damage [183, 184]. Zhang *et al.*, showed that F2 treatment inhibited proliferation of U87 cells by inducing G_2_/M cell cycle arrest and reducing MMP expression while causing little toxicity to normal cells [185]. More recently, Hong *et al.* also showed that F2 inhibits U251 GBM cell invasion and angiogenesis by downregulating hypoxia inducible factor 1 alpha (HIF-1α) mediated MMP-2 and VEGF expression [184]. In addition, F2 also inhibited formyl peptide receptor, a type of G-protein coupled receptor that is known to be intricate in GBM tumor invasion and angiogenesis [185].

### Quercus petraea

*Quercus petraea*, commonly known as the sessile oak, is predominantly found at Walloon forest region in Belgium. Michel *et al.*, discovered that ME of *Quercus petraea* stem bark efficiently inhibit the growth of GBM cell line U373 [186].

### Ochnaflavone

*Ochna kibbiensis* and *Ochna schweinfurthiana* belong to the Ochnaceae family and is distributed in Northern Nigeria. The ethyl acetate extract (EAE) and ME from the leaves of this plant showed cytotoxicity to human GBM cells U-1242, though EAE was more potent than ME [187]. While these results implicate the presence of anti-GBM molecules in *Ochna kibbiensis* [187], its anti-proliferative effects still needs to be investigated.

### Salvia menthaefolia and *Ficus bubu*

*Salvia menthaefolia* is a Chinese herbal medicinal plant from the Lamiaceae family. Giovina *et al.*, found that ME of *Salvia menthaefolia* roots significantly inhibited the viability of the U87, T98G and DBTRG-05MG human GBM cell lines [188]. Similarly, the ME from leaves of another plant *Ficus bubu* (Moraceae family) also inhibited the proliferation of U373 GBM cells [189].

### Auron-misheil-therapy

Auron-Misheil-Therapy (AMT) is a complex mixture comprising of camolile extract (isolated from chamomile blossom) and additives including human insulin, vitamins, calcium and the antihistamine chlorpheniramine. AMT promotes body weight gain and has been used as palliative for pain in the patients with end stage cancers for decades. In addition, recently AMT was shown to inhibit proliferation of U87, LN-229, CNXF 498NL, SF-268 and SF-295 GBM cells along with decreased anchorage independent growth of CNXF 498 and SF-268 GBM cells [190].

## EFFECTS OF NATURAL PRODUCTS ON GBM PRE-CLINICAL MODELS

The anatomical, physiological and genetic make-up of mice relatively recapitulates that of humans [191] and hence they are powerful models to test therapeutic efficacy, investigate pharmacokinetics and characterize acute and chronic toxicities of newly discovered drugs. Here we have summarized all the natural products with anti-tumorigenic effects on GBM mouse xenograft models Table 2.

**Table 2:** Natural products which displayed anti-cancer activities in GBM pre-clinical models

S. No	Scientific Name	Component	Function	Refs
1	*Withania somnifera*	Water extract	↓ tumor volume in rat orthotopic glioma allograft model	[198]
2	*Withania somnifera*	Withaferin A	↑ median survival of orthotopic xenograft mouse model by 40%	[199]
3	*Curcuma longa*	Curcumin	↓ intracranial tumor growth of U87 GBM xenografts and ↑ the overall survival of mice	[201]
4	*Angelica sinensis*	Root chloroform extract	↑ apoptosis in both p53 independent and dependent pathways ↓tumor growth in a rat GBM model & human GBM orthotopic model	[220]
5	*Ardisia pusilla*	Ardipusilloside 1 (ADS-1) polymer microspheres	able to retain ADS-1 release for 36 days in *in vitro* Higuchi model of kinetics; inhibited tumor growth *in vivo* C6 intracranial tumor model and ↑ overall survival of the animal	[224]
6	*Berberis aristata*	Berberine	elicited more cytotoxicity than TMZ in U87, U251 and U118 GBM cell lines; ↓ EGFR-RAF-MEK-ERK signaling and induced senescence; ↓ tumor growth in GBM xenografts	[237]
7	*Anemone taipaiensis*	Saponin 1	↓ expression of survivin, XIAP, Bcl-2/Bax ratio, activating caspase-9, caspase-3 and apoptosis in U251 and U87 GBM cell lines; ↓ tumor growth in U251 and U87 GBM xenografts in mice	[238]
8	*Panax ginseng*	Ginsenoside RG3	↑ TMZ sensitivity, ↓ VEGF-A and BCl-2 in HUVEC and rat glioma cell lines; RG3 and TMZ combinational treatment significantly ↓ angiogenesis.	[244]
9	Iris versicolor	Iridin	↓ intracranial growth of U87 and G144 GBM xenografts in mice	[257]

### Withaferin

*Withania somnifera*, traditionally known as Ashwagandha, has been used in ayurvedic medicine for several thousand years [192]. It contains numerous compounds including 40 withanolides, 12 alkaloids, multiple flavonoids and sitoindosides extracted from different parts of the plant [193, 194]. Among them, Withaferin A appeared to be the most bioactive compound with anti-invasive, anti-angiogenic, anti-inflammatory and pro-apoptotic effects [195]. Grace *et al.* studied the anti-oxidative and anti-inflammatory effects of two withanolide components, namely, Withanolide A and Withaferin A in microglial cells [196]. Surprisingly, withanolide components not only abolished lipopolysaccharide (LPS) stimulated nitric oxide production and ROS generation, they also induced nuclear factor (erythroid-derived 2) like 2 (Nrf2) signaling followed by upregulation of hemeoxygenase-1 (HO-1) [196]. Though both withanolide components displayed anti-oxidative and anti-inflammatory effects, Withaferin A was found to be 10 fold more effective than Withanolide A [196]. In addition, EE of *Withania somnifera* leaves significantly inhibited the proliferation of C6 rat glioma and YKG1 human glioma cell lines in a dose dependent manner [197]. Recently, Patrick *et al.*, demonstrated that Withaferin A treatment significantly decreased the proliferation of U87, U251 and TMZ resistant GBM cell lines U87 TMZ, U251 TMZ, T98G and U138 as well in a concentration dependent manner [53]. Moreover, it increased TMZ sensitivity by downregulating MGMT expression in U251 TMZ, T98G and U138 TMZ resistant GBM cell lines [53]. Interestingly, oral administration of *Withania somnifera* water extract (4 ml/kg/day) significantly reduced the tumor volume in a rat orthotopic glioma allograft model [198]. In addition, an intraperitoneal administration of Withaferin A (12 mg/kg) for 3 days in a week for three weeks also resulted in 40% improvement in median survival of orthotropic xenograft mouse model [199].

### Curcumin

Curcumin, a constituent in turmeric, is derived from the herb *Curcuma longa* that belongs to Zingiberaceae family and used as an ingredient in cooking in some part of India. Curcumin displays significant growth inhibition, prevents angiogenesis and induces apoptosis in *in vitro* and *in vivo* models of many cancers [200]. In GBM, curcumin has been shown to attenuate the intracranial tumor growth of U87 xenografts and increased the overall survival of the mice [201]. Although it’s low bioavailability in humans owing to poor absorption and fast clearance from the body has limited its anti-cancer effects [202, 203], recent research has shown that turmeric rhizomes that are subjected to supercritical (CO2) and hydroethanolic isolation yields an extract known as Turmeric Force™ (TF). The TF contains 11% curcuminoids, 45% turmerones and some other molecules that display more cytotoxicity against cancer cell lines than turmeric [204]. Cheppail *et al.*, also showed that addition of TF to either etoposide or TMZ increased the cytotoxicity in U87 GBM cell line. They further showed that the triple drug combination of TF with etoposide plus TMZ was even more cytotoxic to the U87 cell line [205]. Compellingly, tumerones from TF were also shown to cross the BBB [206]. The extract from the other curcuma species, *Curcuma amada (*CA) also significantly reduced the viability of human embryonal (RD) and alveolar (SJRH30) rhabdomyosarcoma cells [207]. The underlying anti-tumor mechanism showed that CA treatment attenuates AKT signaling, and downregulates anti-apoptotic genes in U87 cells [208]. It is important to mention that AKT is a serine/threonine protein kinase B involved in regulating several biological functions including cell proliferation and invasion, apoptosis inhibition, angiogenesis [209], and chemotherapeutic resistance [210–213]. Activated AKT is known to inhibit apoptosis by abolishing the cytochrome-c release from the mitochondria and inactivating pro-apoptotic proteins procaspase-9 and BAD by phosphorylation [214, 215]. Further, no significant effect on the control mouse hypothalamus cell line (mHypoE-N1) suggests that CA may be nontoxic to normal brain cells.

### Angelica sinensis

*Angelica sinensis,* commonly known as dong quai or “female ginseng”, is an herb from the family Apiaceae. It is acknowledged for its efficacy in treating gastric mucosal damage, chronic glomerulonephritis and diminished myocardial blood flow [216–219]. *Angelica sinensis* root chloroform extract treated GBM cell lines undergo apoptosis in both p53 independent and p53 dependent pathways [220]. It curbed tumor growth in a rat GBM model, and in a GBM orthotopic model, suggesting that it might pass through the BBB [220].

### Ardipusilloside 1

Ardipusilloside 1 (ADS-1) is a triterpenoid saponin, extracted from the medicinal herb *Ardisia pusilla* with well known anti-cancer activity on GBM cell lines [221, 222]. Unfortunately, a short half-life in plasma (5.61 h) of ADS-1 due to deglycosylation [223] limits its therapeutic efficacy *in vivo*. However, recently developed ADS-1 polymer microspheres packed into wafers [224] have increased its half- life to 36 days in *in vitro* and *in vivo*. In animal models, these polymers significantly reduced the C6 intracranial tumors and increased overall survival [224]. Mechanistically, the decrease in tumor growth was due to decreased tumor necrosis factor -α, interleukin-6, C-reactive protein, VEGF and upregulation of interleukin-2 expression [224].

### Toosendanin

Toosendanin (TSN) is a triterpenoid saponin isolated from the Chinese herb *Melia toosendan* and is known to inhibit acetylcholine release at the nerve terminals. Though used as a pesticide, recent studies have shown its anti-proliferative effect against lymphoma, leukemia and hepatocellular cancer cell lines [225–228]. In addition, TSN also inhibits proliferation of U87 and C6 cell line at nanomolar (10 nM) concentrations [229]. Additionally, TSN reduced the growth of U87 xenografts *in vivo* feasibly by upregulation of p53 and estrogen receptor β, which are known tumor suppressors in many cancers [229–231].

### Berberine

The alkaloid berberine is extracted from the stem bark, rhizome and root of several Chinese medicinal plants including *Berberis aristata*, *Berberis aquifolium* and *Tinospora cordifolia* [232, 233]. Berberine shows significant antihelmintic, anti-inflammatory, antimicrobial and antioxidative properties [234, 235]. Ki *et al.* demonstrated apoptosis inducing effects of berberine on human GBM T98G cells in a concentration dependent manner [236]. Furthermore, berberine induced cell death was coupled with increased ROS production, intracellular calcium levels and endoplasmic reticulum (ER) stress [236]. A recent study showed more cytotoxicity of berberine in U87, U251, and U118 GBM cells than by TMZ [237]. They showed that berberine induced senescence was associated with EGFR-RAF-MEK-ERK signaling pathway inhibition. They further reported growth inhibition of GBM xenografts using berberine [237].

### Saponins

*Anemone taipaiensis* from the Ranunculaceae family is a traditional Chinese medicine used against rheumatism and phlebitis. Recent studies demonstrated that saponin-1 and saponin-B from the A*nemone taipaiensis* inhibited the growth of brain tumor *in vitro* and *in vivo* [238, 239]. Mechanistic studies revealed that saponin-B induced apoptosis in U87MG cells was associated with cell cycle arrest in S phase, activation of Fas-l, increased Bax/Bcl-2 ratio and caspase-3 activation [239]. Similarly, saponin-1 was shown to decrease survival and induce apoptosis in U251MG and U87MG cells by decreasing the expression of survivin, XIAP, Bax/Bcl-2/ ratio and activation of caspase-9/3 [238]. Moreover, Li *et al.* also showed that saponin-1 inhibited the growth of U251MG and U87MG xenografts in nude mice [238]. Similarly, saponin-6 also induced cell cycle arrest and apoptosis in U87 MG cells by DNA fragmentation, increasing caspase 3/9 activity, increasing expression of Fas and Fas ligand, and decreasing Bcl2 expression [240]. In a comparative analysis, Xiaoyang showed that oleanane type saponins display more cytotoxicity to U87 GBM cells than to benign human lung (A549), hepatocellular (HepG2), cervical (Hela) and human promyeleocytic leukemia HL-60 cell lines [241].

### Ginsenoside RG3

Ginsenoside RG3 is mainly extracted from *Panax ginseng* (or ginseng) that is widely used as a medicinal plant in Asia [242]. Ginsenosides are triterpenoid saponins and ginsenoside RG3 comes under protopanaxatriols group, in which sugar moieties are linked to the β-OH group at C-20 or C-3 [243]. Caixing *et al.* showed that ginsenosides RG3 increased TMZ sensitivity in C6 rat glioma cells, downregulated VEGF-A and BCl-2 and induced apoptosis in HUVEC [244]. In addition, ginsenosides RG3 combined with TMZ also significantly reduced angiogenesis [244].

### Natural products on GBM Cancer Stem Cells

CSCs also termed tumor initiating cells are a very small population of cancer cells that are responsible for the tumor initiation and resistance to CRT [245–247]. In GBM, CSCs are distributed in a specialized location known as the perivascular niche (PVN), which also harbors endothelial cells, astrocytes, tumor cells, microglia and pericytes [248, 249]. The PVN is presumed to maintain stemness and thereby the resistant nature of the CSCs [250–253]. While conventional CRT has been shown to enrich the CSC population and promote tumor recurrence, natural products may better target CSCs in multiple cancer types including GBM [254–256]. Rajarshi *et al.*, identified three plant based non-toxic natural compounds from the Microsource Spectrum Collection library (MSCL)– namely, Iridin, triacetyl-resveratrol (TAR) and tigogenin–which specifically target the PVN [257] and inhibit the intracranial growth of U87 and G144 GBM xenografts in mice [257], suggesting that additionally targeting the PVN may improve survival of GBM patients.

CSCs that are isolated from GBM8401 cells are resistant to the cytotoxic drug TMZ [258]. MSC500 is a Korean natural medicine recipe consisting of eight herbs including *Gastrodia elata, Phellinus linteus,* and *Mulberry* leaves. Interestingly, MSC500 treatment to the GBM8401 cells resulted in a dose dependent decrease in aldehyde dehydrogenase (ALDH) activity associated with a reduction of the CSC population [258]. In addition, it downregulated the expression of ABC transporters (ABCG2 and ABCB5) in GBM8401 CSCs, known to impart drug resistance [258]. While a recent study showed decreased CSC population (from 3.6 % on day 3 of treatment reduced to 0.55% on day 10) by curcumin in the C6 rat glioma cells [259]; its encapsulation using a biodegradable nano-carrier dentrosome (DC) also reduced the growth of U87 cells and decreased the expression of pluripotency genes like Nanog, SOX2, OCT 4A & 4B1 [260, 261]. DC also increased the expression of tumor suppressor miRNA-145, which is usually downregulated in several tumor tissues [260, 261]. It is noteworthy that polyurethane-short branch polyethylenimine vehicle mediated miRNA-145 delivery in CSCs abolished their stemness [262]. Other studies showed that by inhibiting PI3K/AKT/NF-kB signaling and decreasing MMP-2 expression, resveratrol inhibited GBM CSCs invasion in both *in vitro* and *in vivo* models [263]. Similarly, eckol, a phlorotannin compound extracted from *Ecklonia cava* with known anti-oxidant activity [264, 265] was shown to suppress the stemness in U87 and U373 GBM cells and in GBM patient derived CSCs XO1 GB and XO3 AOA by inhibiting PI3K/Akt and Ras/Raf-1/Erk signaling [266]. In another study, eckol was also shown to abolish the anchorage independent growth of U373 glioma cells and growth of xenografts [266]. It is not yet clear if the mechanism of action for this effect on CSCs was mediated through ROS generation.

### Natural products and Blood Brain Barrier

As GBM is highly invasive in nature, resection of all microscopic disease is not practically feasible, even when tumor is in non-eloquent regions. Though targeted therapy has attracted much attention, it has not increased the overall survival (OS) of GBM patients, due partly to the poor penetration of drugs through the BBB. Therefore, agents capable of modulating the BBB permeability to improve the bioavailability of therapeutic drugs to the tumors are highly desirable.

The BBB is a complex cellular vascular structure that by paracellular or transcellular pathways regulates the access of molecules to the brain. It is composed of numerous tight junctions between endothelial cells, ATP dependent multidrug resistance (MDR) pathway proteins known as P-glycoprotein (P-gp), enzymes, and receptors [267]. While the phosphorylation of endothelial tight junctional (TJ) proteins, occludin or zonula occludens-1 (ZO-1) regulate the paracellular permeability of the BBB [268], pharmacological inhibition of P-gp can increase drug influx to the brain [269]. The BBB microenvironment that includes astrocytes, microglia, pericytes, neurons, fibroblasts, basement membrane, extracellular matrix (ECM) and adjacent cell types [270], also influence BBB functions [271, 272]. The BBB of tumors (BBB) originates from tumor capillaries supplying nutrients and oxygen to the tumor [273]. The glioma BBTB microenvironment consists of tumor cells, ECM, tumor -associated microglia, infiltrating macrophages and other cell types. Interestingly, targeting the glioma microenvironment suppressed tumor growth in rat GBM models [274].

**Figure 2:**
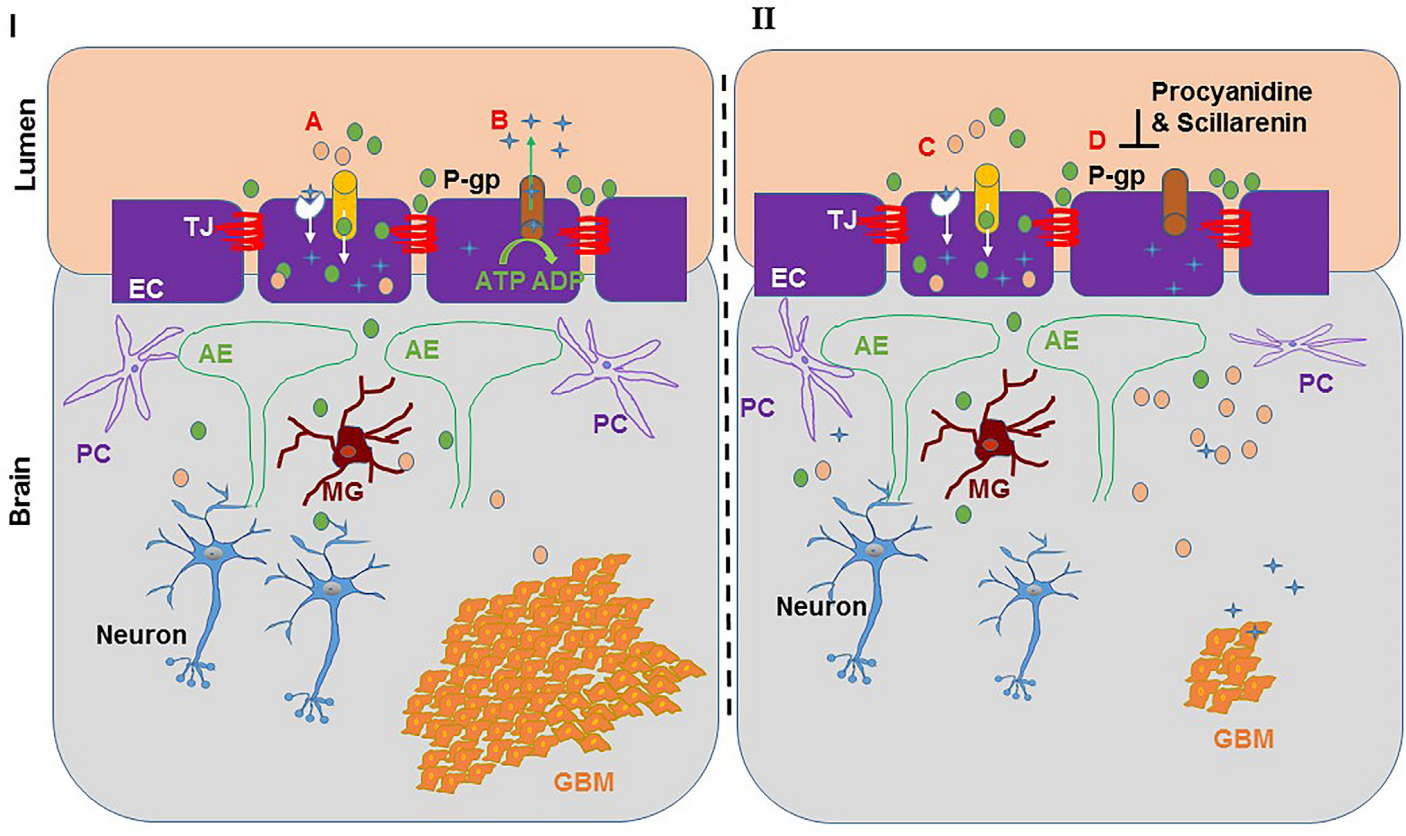
Natural products modulating blood brain barrier permeability Natural products aid the permeation of chemotherapeutic drugs in the brain. On the left of the diagram **I**: A- shows the influx of sugars and amino acids from the blood to the brain by a selective nutrient transporter in the endothelial cells, identified as solute carrier proteins and passive diffusion of chemotherapeutic lipophilic drugs; B- showing P-gp present in the endothelial cells pumps out most drugs in a ATP dependent manner and lowers the drug concentrations in the brain. On the right side of diagram **II**: C & D- shows natural compounds like procyanidine and scillarenin inhibit P-gp protein thereby allow accumulation of drug (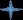) in the brain to reduce the tumor burden. TJ, Tight junctions; EC, endothelial cells; P-gp, P-glycoprotein; PC, pericytes; AE, astrocytic endfoot; MG, microglia.

Natural products are known to modify the BBTB microenvironment through modulating the function of endocytosis, P-gp and secretion of MMPs. Shikonin, the prominent naphthoquinone isolated from a medicinal herb *Lithospermum erythrorhizon*, is known for its anti-oxidant and anti-inflammatory activities [275–277]. Lina *et al.*, found that shikonin treatment significantly suppressed MMP-9 expression while it increased claudin-5 and BBB permeability in mice after ischemic stroke [278]. Additionally, shikonin treatment was found to reduce the viability, migration and invasion of the GBM cell lines U87 and U251 and also to decrease the expression of MMP-2 and MMP-9, conceivably through the inhibition of PI3K/Akt signaling [279]. Haidong *et al.*, observed that resveratrol treatment protects the integrity of the BBB by regulating expression and activities of MMP-9 and TIMP-1 in rat brains that were re-perfused after ischemic insult [280]. Similarly, procyanidine from the bark of *Pinus massoniana* also modulated microvessel endothelial cell permeability perhaps by modulating P-gp proteins in a rat brain model. More interestingly, procyanidine increased adriamycin permeation through BBB resulting in enhanced therapeutic efficacy and increased overall survival of mice [281]. In addition, Scillarenin from scilla [272], has also been shown to prevent P-gp mediated efflux [282] (Figure 2). Yan-feng *et al.* also demonstrated that during hypoxia and glucose deprivation, curcumin maintains the BBB integrity by regulating ZO-1 and occludin expression [283].

## SUMMARY AND CONCLUSION

GBM, the most common malignant brain tumor in adults, remains incurable with a bleak median survival. Despite multiple efforts there have been very few FDA approved drugs for its treatment, which are not universally efficacious. The efficacy of TMZ, a DNA alkylating agent used in first line therapy, is weakened by the expression of MGMT, which repairs the DNA damage induced by TMZ. Interestingly, multiple compounds from natural sources namely resveratrol, icariin, quercetin, propolisis, Turmeric Force™ and Withaferin A work synergistically with TMZ [28, 32, 33, 37, 49, 53, 205]. *Zataria multiflora* hydroalcoholic extract and Tet also significantly increased the radiosensitivity of the A172 and the U87 and U251 GBM cell lines respectively [86, 88]. Resveratrol, *Withaferin A*, quercetin, methanolic extract of *Salvia menthaefolia* roots, berberine, *Ficus carica* latex, propolisis, AMT, thymoquinone and cucurbitacins efficiently blocked cell proliferation and induced apoptosis, even in the TMZ resistant GBM cell lines [27, 32, 42, 49, 53, 113, 125, 188, 190, 236]. Moreover, TMZ induced cytotoxicity may be modulated by wild type p53 status. It has been observed that siRNA mediated silencing of p53 confers resistance to TMZ [284]. Toosendanin from *Melia toosendan* increased the p53 expression [229] and betulinic acid from *Betula pubescens*, quercetin, diosquinone from *Diospyros tricolor*, chloroform extract of *Angelica sinensis* root significantly induced cell death even in p53 mutated cell lines [29, 132, 164, 220]. Interestingly, administration of Withaferin A, curcumin, *Angelica sinensis* root chloroform extract, Ardipusilloside 1 and berberine significantly reduced the tumor volume and increased the overall survival in an orthotopic GBM animal model [199, 201, 220, 224, 237], suggesting that these compounds may cross the BBB. Natural compounds resveratrol, curcumin, eckol and Korean natural medicine recipe MSC500, significantly eradicated the CSC population in GBM [258, 259, 263, 266] and decreased the *in vivo* tumor growth in mice xenografts [266]. Most chemotherapy trials have failed in GBM patients, due partly to the poor penetration of drugs through the BBB. Natural products may modify the BBB permeability by altering the function of its components. In particular, procyanidine extracted from *Pinus massoniana,* was found to inhibit P-gp and increased the therapeutic efficacy of adriamycin by allowing it to permeate the BBB in nude mice, increasing their overall survival [281].

The few clinical trials evaluating the use of natural products for GBM have been relatively underpowered. Galactoside-specific lectin from mistletoe (ML-1) plant extract has immunoprotective/immunostimulatory activity [285–287]. Addition of ML-1 to the standard treatment for grades III and IV astrocytoma patients significantly increased the overall survival (20.05 ± 3.5 Vs 9.90 ± 2.1 months) [288]. Patupilone (epothilone B), a microtubule-stabilizing natural cytotoxic compound with BBB permeation and long half-life displayed progression free survival in recurrent GBM patients [289, 290].

As summarized here, a wealth of preclinical data exists to support further study using natural products in GBM. This dismal prognosis demands that we explore alternative therapy to improve outcomes for these patients. Prospective randomized clinical trials must be done to explore the use of adjunctive natural therapy in better targeting resistance and synergistically improving upon standard treatments.

## Abbreviations

ALDH: Aldehyde dehydrogenase
BBB: Blood Brain Barrier
BBTB: BBB of tumors
CDKs: Cyclin-dependent kinases
CNS: Central Nervous System
CRT: Chemo-radiation Therapy
CSCs: Cancer Stem Cells
CT: Chemotherapy
ECM: Extracellular matrix
ER: Endoplasmic reticulum
ERK: Extracellular signal-regulated kinase
GBM: Glioblastoma
GFAP: Glial Fibrillary Acidic Protein
Hsp-27: Heat shock protein 27
HIF-1 α: Hypoxia Inducible Factor 1 alpha
HO-1: Hemeoxygenase-1
IL-6: Interleukin - 6
JAK: Janus Kinase
LGR5: leucine-rich repeat containing G-protein coupled receptor 5
LPS: Lipopolysaccharide
MAPK: Mitogen Activated Protein Kinase
MDR: Multidrug resistance
MGMT: O6-methylguanine-DNA methyl transferase
miRNAs: MicroRNAs
MMP: Mitochondrial Membrane Potential
MMPs: Matrix metalloproteinases
NPC: Nuclear Pore Complex
Nrf2: Nuclear Factor (erythroid-derived 2) like 2
OS: Overall survival
P-gp: P-glycoprotein
PI3K: phosphoinositide 3-kinase
PVN: Perivascular niche
RIP: Ribosome Inactivating Proteins
ROS: Reactive Oxygen Species
RT: Radiotherapy
RTK: Receptor Tyrosine kinase
STAT-3: Signal Transducer and Activator of Transcription- 3
TGF-β1: Transforming growth factor - β1
TJ: Tight Junction
TMZ: Temozolomide
VEGF: Vascular Endothelial Growth Factor
XIAP: X-linked inhibitor of apoptosis protein
